# ROBECS one: feasibility study of a structured robotic curriculum and a proficiency-based framework

**DOI:** 10.1007/s11701-026-04136-4

**Published:** 2026-11-03

**Authors:** Meret Appel, Carla Sacher, Lucien Cron, Amanda Dirnberger, Beat P. Müller, Jennifer M. Klasen

**Affiliations:** 1 Department of Visceral Surgery, Clarunis, University Digestive Health Care Centre Basel, St. Clara Hospital and University Hospital Basel, Basel, Switzerland; 2 Faculty of Medicine, University of Basel, Basel, Switzerland

**Keywords:** Robotic surgery, Surgical education, Proficiency-based progression, Simulation-based training, Surgical curriculum design

## Abstract

Robotic surgery is increasingly integrated into surgical practice, yet structured, benchmark-driven simulation training remains limited for general surgery trainees. Proficiency-based progression (PBP) offers a practical, specialty-tested approach for structuring robotic surgical training around demonstrated performance rather than case numbers, but standardized PBP curricula for general surgery robotic training remain underdeveloped. This study aimed to test the feasibility of a structured, simulation-based robotic training and, building on this, to develop a preliminary proficiency-based approach whose benchmarks can be formally evaluated in future studies. We conducted a prospective, single-center pilot feasibility study of a structured robotic simulation curriculum over two weeks, combining virtual reality simulation and dry-lab training on the da Vinci Xi system (Intuitive Surgical, Sunnyvale, CA, USA). Advanced and novice robotic surgeons completed stepwise training sessions targeting core robotic skills and procedural tasks. Feasibility was defined by successful implementation within the timeframe, completion of sessions, and participant acceptance, assessed informally; the curriculum was subsequently structured into skill modules with proposed proficiency benchmarks. The curriculum was successfully implemented within the planned two-week timeframe, with six advanced and fifteen novice robotic surgeons participating. The curriculum was structured into six sequential modules, representing key technical and non-technical skills in robotic surgical practice, each with proposed proficiency benchmarks. This pilot study demonstrates the feasibility of a structured robotic simulation curriculum in general surgery. The proposed benchmark-driven structure may inform the development of standardized robotic simulation curricula for general surgery training programs, pending further validation against clinical performance.

## Introduction

Robotic surgery has become an integral component of modern surgical practice across multiple specialties. Initially established in urology, the adoption of robotic platforms has expanded rapidly over the past two decades to include general surgery, gynecology, cardiothoracic, and neurosurgery [1–3].

The technological advantages of robotic systems, including enhanced three-dimensional visualization and improved instrument articulation, enable precise minimally invasive procedures and have been associated with favorable short-term clinical outcomes in selected settings [3–5].

Despite these advances, the increasing use of robotic technology in surgery has introduced new challenges for surgical education [6–8]. These challenges compound broader pressures already facing surgical training, including regulatory restrictions on working hours and evolving expectations of trainees, which have strained traditional models and prompted calls for more structured, resource-efficient curricula across surgical specialties [7, 9–11]. Robotic surgery brings these challenges into particularly sharp focus, as trainees must acquire an additional, technically distinct skill set within an already time-constrained training period. Traditional surgical education models based primarily on case numbers are recognized to be insufficient to ensure safe and efficient skill acquisition [12]. Consequently, there is a growing need for structured, benchmark-driven pathways that define how proficiency is acquired, objectively assessed, and progressively advanced [13, 14]. Such approaches are particularly relevant in robotic surgery, where increasing procedural complexity and technological advancement demand more deliberate and standardized pathways to competence [1, 15].

Proficiency-based progression (PBP) offers a practical approach for structuring robotic training around demonstrated, benchmarked performance rather than case numbers or time served [12, 13].

A central element of PBP is objective, metric-based benchmarking: training advances only once a trainee has repeatedly demonstrated performance at or above a predefined proficiency threshold, rather than after a fixed number of repetitions or a supervisor’s subjective impression [12, 13]. Specific benchmarks should be based on the objectively assessed performance of experts performing the target tasks [16]. Validated assessment tools, such as the Global Evaluative Assessment of Robotic Skills (GEARS), are commonly used to operationalize these benchmarks in robotic and laparoscopic surgery training [14, 17].

Robotic surgery appears particularly well suited to a proficiency-based approach because it involves clearly delineated technical skill sets, such as robotic system setup, console operation, tissue handling, and team communication, that can be decomposed into discrete, measurable tasks. Simulation-based training can support the acquisition and objective assessment of these skills before their application in the operating room.

Several multimodal robotic training curricula have been developed to address this need. Programs, such as the Robotic Surgery Training Curriculum (RoSTraC), combine virtual reality (VR) simulation, dry-lab practice on synthetic organ models, and gradual integration into the operating room, with demonstrated improvements in GEARS scores and successful trainee incorporation [4, 17–19]. Consensus frameworks such as the European Robotic Urology Standard (ERUS) curriculum call for defined proficiency benchmarks, protected training time, and case-log tracking [1, 14]. Similar structures have recently been proposed across general and colorectal robotic surgery [20, 21]. Recently, a large international Delphi consensus study established a platform-agnostic robotic training curriculum for gastrointestinal surgical trainees, structured around e-learning, proficiency-based simulation, bedside assisting and live case observation, and supervised console operation with objective, metric-based sign-off [22]. However, standardized, benchmark-driven simulation curricula for general surgery trainees remain limited, particularly outside high-volume specialty centers.

Therefore, the primary aim of this study was to explore the feasibility of a structured, simulation-based robotic training course within a clinical training environment. Feasibility was defined as successful implementation within the planned timeframe, completion of all training sessions, and participant acceptance of the curriculum, assessed through written participant feedback. Building on the feasibility, a secondary aim was to develop a preliminary proficiency-based approach, structuring the curriculum into training modules with defined benchmarks that can be formally evaluated in future studies.

## Materials and methods

This prospective pilot feasibility study evaluated a structured, simulation-based robotic training program at the Department of Visceral Surgery, Clarunis University Digestive Health Care Centre Basel, Switzerland. The program, Robotic Educational Curriculum for Surgeons, called ROBECS One, was implemented over a two-week period in May and June 2025. The duration of this pilot program was determined by the period during which a da Vinci Xi system with an integrated simulation platform was provided by Intuitive Surgical (Sunnyvale, CA, USA). Surgeons from the department were invited to participate and were assigned to one of two groups according to their previous robotic experience.

All advanced robotic surgeons were board-certified and had previous robotic experience as independent console surgeons. Novice robotic surgeons were residents without prior experience as a console surgeon; some of them had initial experience as bedside assistants. Advanced robotic surgeons were included to test the full curriculum and judge its content and sequence in relation to clinical requirements. Novice surgeons represent the population for whom the curriculum is ultimately intended. All training sessions were planned during protected working hours.

Following the design and implementation of the curriculum, we subsequently sought to structure the training program into modules with defined proficiency benchmarks, following a proficiency-based progression approach. These benchmarks were not formally validated within this study and were developed with the intention of being validated within future research.

### Curriculum development and structure

ROBECS One was designed as a structured, multimodal training program consisting of three sequential phases: foundational training in basic robotic skills on the VR simulator, procedure-oriented simulations, and dry-lab training on synthetic organ models. The selection and sequencing of training components were guided by key competencies required in robotic visceral surgery [14]. Prior to beginning training, the surgeons completed an organized in-service orientation and introductory online module to familiarize themselves with the basic principles of the simulation program. Training sessions were conducted in peer-teams of surgeons of the same experience level: advanced surgeons trained in pairs with a four-hour time slot per phase, and novice surgeons in teams of three to four with two-hour sessions. The peer-team training format allowed efficient use of limited console and simulator time and, for the advanced pairs, corresponds to the dyad format described in laparoscopic training, with evidence showing comparable skill acquisition to individual training with reduced procedure time [23].

Feedback on the curriculum structure, sequence and its learning opportunities was collected informally in writing by one author (M.A.) after completion of the training, as unstructured comments without a questionnaire. The comments were summarized descriptively.

### Curriculum for advanced robotic surgeons

After completion of the in-service training and introductory online module, the in-person training program followed a stepwise structure over five to seven days. Each peer-team attended one training session per day, each lasting four hours. The curriculum was organized so that foundational simulator exercises were performed on day one to develop basic console skills, followed by procedure-oriented simulations in a subsequent session, and dry-lab training on a final dedicated day. The three phases were thus scheduled on separate days, typically with intervals of one to two days between sessions.

In the initial phase, fundamental console exercises using the SimNOW platform from Intuitive Surgical were performed with the aim of covering all key console skills such as clutching, camera navigation, instrument articulation, multiple-arm control, usage of energy pedals and performance of a running suture. These exercises were repeated three times per session by each surgeon before progressing to the next phase. The number of repetitions was fixed in advance and was not linked to a performance threshold. The participation of the advanced robotic surgeons served primarily to test the feasibility, content and sequence of the curriculum.

**Fig. 1:**
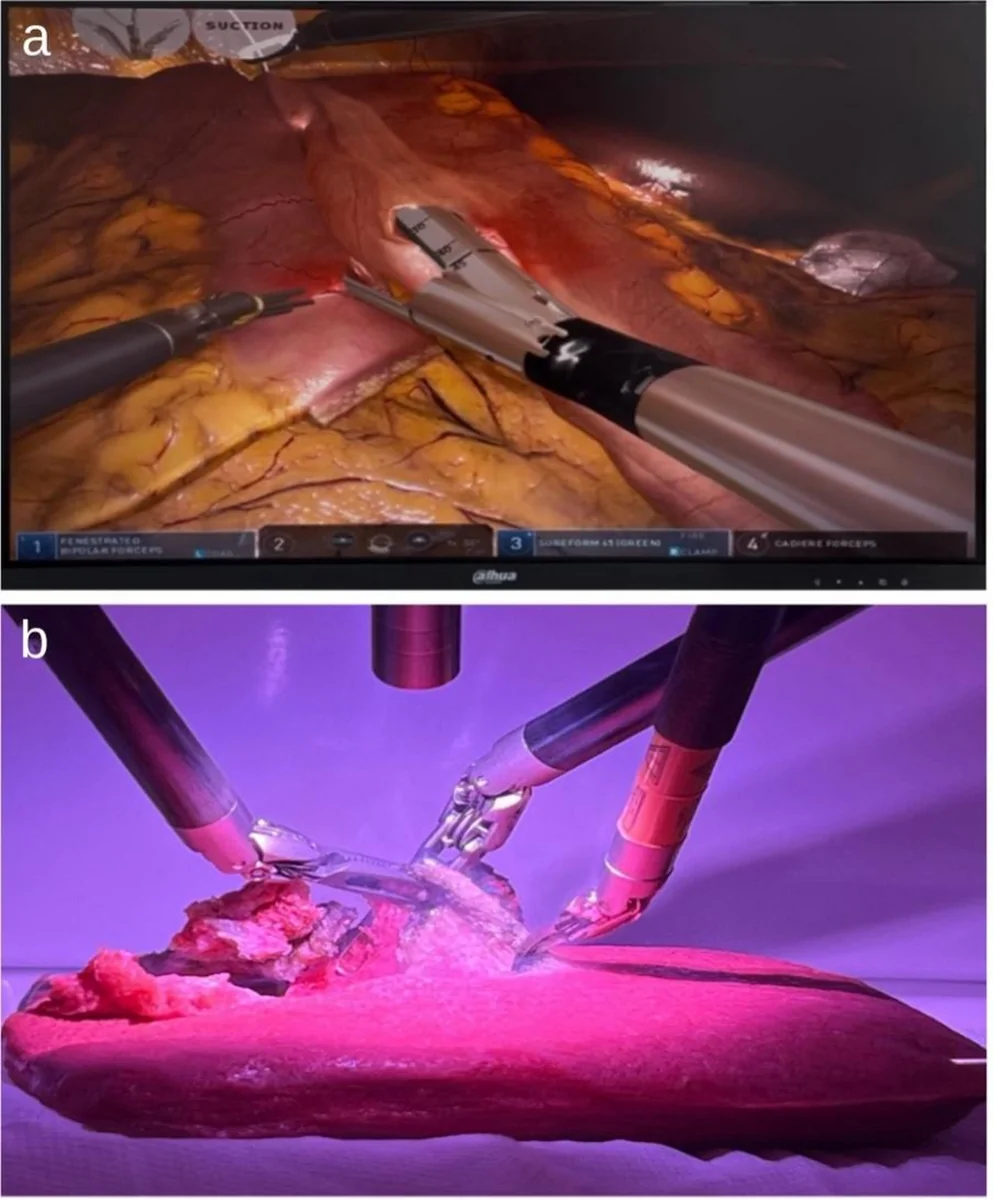
(**a**) Simulated right hemicolectomy with intracorporeal anastomosis, (**b**) Dry lab exercise on synthetic organ models

In phase two, the advanced robotic surgeons progressed to procedure-based simulations, alternating between first and second operator roles to practice coordinated use of multiple robotic arms, camera control, dissection, and suturing in a more realistic procedural context. Two procedure modules were selected: one representing a less complex, commonly performed operation (cholecystectomy) and one representing a more advanced intervention (right hemicolectomy with intracorporeal anastomosis) (Fig. 1a).

In phase three, dry-lab exercises were carried out on synthetic human organ models to further consolidate tissue-handling skills, visual-spatial perception and force-translation. These dry-lab tasks focused on intracorporeal suturing and knot tying on artificial abdominal organs (Fig. 1b). Simulator-derived performance scores were displayed for immediate individual feedback but were not recorded for analysis.

### Curriculum for novice robotic surgeons

Following the completion of the curriculum for advanced robotic surgeons, a shortened training phase with identical structure was offered to the novice robotic surgeons. They completed the shortened program over three days, scheduled within one of three available daily time slots, in teams of three to four surgeons with sessions lasting two hours, thereby gaining initial exposure to robotic simulation training.

### Proficiency benchmark development

Following curriculum implementation, we aimed to structure the curriculum into modules and corresponding proficiency benchmarks.

These modules and benchmarks were defined around key technical and non-technical tasks within robotic surgical practice and subsequently refined through a review of the literature. Simulation-based training can be used as a structured educational environment in which trainees can work toward these benchmarks under standardized, repeatable conditions.

The selection and structure of these benchmarks were informed by proficiency-based progression principles established in prior robotic and laparoscopic skills curricula [12–16, 22, 24]. The proposed benchmarks were not formally validated within this study and were intended to serve as a preliminary framework with the goal to inform future research.

## Results

The robotic simulation curriculum was successfully implemented within the planned timeframe. The training was performed on a da Vinci Xi robotic system with an integrated VR simulation platform (SimNOW), provided temporarily by an industry representative from Intuitive Surgical.

A total of six advanced and fifteen novice robotic visceral surgeons participated. Their characteristics are summarized in Table 1. The training program was successfully implemented within the available clinical schedule, demonstrating feasibility within a clinical training environment.

**Table 1:** Characteristics of the participating surgeons

**Advanced robotic surgeons (** ***n*** ** = 6)**	
Years of independent practice since board-certification, median (range)	5 (3–9)
Robotic procedures as a console surgeon, median (range)	26 (5–65)
**Novice robotic surgeons (** ***n*** ** = 15)**	
Postgraduate years, median (range)	3 (1–6)
Cases as a robotic bedside assistant, median (range)	6 (0–24)

We structured the curriculum into six sequential modules, each targeting a defined set of technical or non-technical skills within robotic surgical practice, with corresponding proficiency benchmarks (Table 2). Module 1 focuses on the foundational understanding of robotic system setup, enabling trainees to safely prepare and manage the robotic platform. Module 2 addresses the role of the bedside assistant, including patient-side instrument exchange and coordination with the console surgeon. Module 3 targets the safe and coordinated operation of the robotic console, encompassing camera navigation, instrument control and multi-arm coordination. Module 4 focuses on tissue handling, dissection and suturing, including visual-spatial perception and force translation, which are critical for safe and effective robotic surgery. Module 5 is an integrative station in which trainees combine console operation and tissue-handling skills to perform a complete, straightforward robotic procedure. Finally, Module 6 expands the scope to non-technical and situational skills, requiring trainees to manage intraoperative robotic system issues and engage in effective team communication.

Together, these modules describe a progression of technical and non-technical skills relevant to robotic surgical practice, from system preparation to procedural performance and management of intraoperative challenges. The simulation curriculum described here provides a structured environment in which trainees can work toward these benchmarks in a safe setting before their application in the operating room. Because trainee performance data were not collected in this pilot, the proposed proficiency benchmarks (Table 2) were adapted from published robotic and laparoscopic proficiency-based curricula [12–16, 22, 24] and matched to the type of skill involved: simulator-derived performance metrics and GEARS for continuous, psychomotor console-based skills, structured direct-observation checklists for discrete, safety-critical procedural tasks such as setup and bedside assistance, and procedure-specific or non-technical skills tools for whole-procedure performance and team-based competencies, respectively.

As the participant feedback was collected as unstructured written comments, it is reported descriptively and was not quantified. Feedback from the advanced participants was predominantly positive. Participants particularly valued the stepwise progression from console-based exercises to procedure-based simulation and dry-lab practice, the protected training time, and the peer-team format, which was felt to reduce time pressure, facilitate mutual learning and allow participants to benefit from on-site instruction provided by the device manufacturer. No participant reported the training as burdensome or of limited relevance. Constructive comments related mainly to session structure and logistics, such as a mandatory introductory simulator session before console access tailored to prior experience level, a wish for additional simulator time within each four-hour session, and an interest in a broader range of procedure-based modules in future iterations. Advanced surgeons also noted that simulator accounts were not linked to the manufacturer’s cloud-based platform, limiting the ability to track individual training progress. Participants did not raise concerns regarding the safety, feasibility, or clinical relevance of the curriculum content.

Feedback from the novice participants, collected in the same informal manner, was also predominantly positive. Trainees described the curriculum as providing a good overview of robotic surgery together with detailed theoretical background and valued the opportunity to try out the different components of the system without time pressure. The most frequently expressed wishes for improvement were for additional console time and a greater number of practical exercises within the curriculum.

**Table 2:** Modules, component skills, assessment methods, and proposed proficiency benchmarks for robotic surgery training

Modules	Component skills	Assessment method	Proposed proficiency benchmark
Module 1: System setup, docking, and troubleshooting	Safe entry, docking, and undocking of the robotic system; recognition and troubleshooting of technical/system errors; patient positioning and system preparation for a planned procedure	Structured direct-observation checklist	Completion without critical error, within a locally defined target time, on direct observation checklist
Module 2: Bedside assistance	Instrument exchange; troubleshooting at the patient side; emergency undocking; coordination with the console surgeon	Structured direct-observation checklist	Satisfactory checklist assessment with zero critical safety errors, across at least two consecutive simulated attempts
Module 3: Console operation	Camera navigation; clutching and instrument repositioning; multi-arm control and switching; instrument articulation; energy pedal use	Simulator-derived performance metrics (time, economy of motion, error rate, instrument collisions) and GEARS	GEARS composite score at or above the mean score of experienced robotic surgeons, sustained across at least two consecutive attempts, with an instrument-collision/error rate below the expert-derived threshold
Module 4: Tissue handling, dissection, and suturing	Tissue handling and dissection; suturing and knot tying; management of visual-spatial perception and force translation	VR and dry-lab simulator task scores	Task scores at or above expert-derived proficiency levels, with acceptable economy of motion and safe energy application
Module 5: Whole-procedure performance (integrative)	Integration of console operation and tissue-handling skills into performance of a complete, basic robotic procedure (for example cholecystectomy)	Procedure-specific assessment tool	Procedure-specific tool score at or above expert-derived threshold, procedure completed within a target time, without major simulated adverse events
Module 6: Non-technical skills and team communication	Situational awareness; team communication; management of intraoperative system issues and unexpected events	Structured non-technical skills assessment	Satisfactory rating during simulated crisis or team-communication scenarios, as judged by a trained observer

## Discussion

This pilot study demonstrates the feasibility of implementing a structured robotic simulation curriculum within a clinical training environment. In addition, we structured the curriculum around a set of proficiency benchmarks for robotic surgery and outlined how simulation-based training could support surgeons in progressing toward these benchmarks. This approach follows the proficiency-based progression (PBP) paradigm that has been increasingly adopted across robotic surgery subspecialties [1, 12, 13, 16, 22, 25]. Robotic surgery is particularly well suited to this approach, since its core tasks, system setup, console operation, tissue handling, and team communication, can be decomposed into discrete, individually observable and measurable skills that can be practiced repeatedly and benchmarked objectively, aligning closely with the logic of proficiency-based progression training described for other robotic and laparoscopic skill sets [13, 15].

A key strength of this approach is that the proposed benchmarks are defined by predefined, objective performance criteria matched to the type of skill, rather than by subjective judgment or case counts alone. Surgical trainees follow their individual learning curves and require varying amounts of training to achieve proficiency. Nevertheless, most surgical training programs continue to rely primarily on case numbers to evaluate readiness for independent practice, despite substantial variability in individual learning trajectories [12]. Proficiency-based progression offers an alternative, focusing on demonstrated, benchmarked performance rather than exposure alone, and allowing for more individualized and potentially more efficient advancement through training [12, 13]. Non-technical skills are equally important in this context. Robotic surgery introduces new challenges due to the physical separation of the surgeon at the console, requiring effective team communication, coordination and situational awareness. Integrating these skills into proficiency-based training is therefore essential for safe robotic practice.

Simulation-based training provides an important opportunity to acquire and assess both technical and non-technical skills before exposure to the operating room, establishing a baseline level of proficiency and potentially enhancing patient safety during the early phases of robotic training [14, 15]. Translation of these benchmarks into formal clinical entrustment decisions, the process by which supervisors grant a trainee a defined level of supervised or unsupervised responsibility for a clinical task [26], was beyond the scope of this pilot. Instead, simulation provides a structured, low-stakes environment in which skills can be practiced and assessed before clinical application, supporting a stepwise transition from simulation-based training to independent operative practice. Structured proficiency benchmarks, such as those proposed in Table 2, also offer the potential to harmonize training across institutions by defining clear, comparable performance thresholds for advancement.

ROBECS One shares conceptual features with the multimodal curricula introduced in the Introduction, combining VR simulation, dry-lab training, and structured skill progression in a manner comparable to RoSTraC and the international Delphi consensus framework [4, 22]. Unlike these longer-term, longitudinal programs, the current pilot condensed training into a short, intensive two-week block designed primarily to test feasibility within routine clinical operations, with formal benchmark validation planned for future iterations. This alignment with a growing body of proficiency-based frameworks offers a practical starting point for building structural robustness in subsequent program iterations, and may contribute to more standardized, benchmark-driven robotic training pathways.

The simulator exercises used in this pilot were taken from the existing SimNOW library, and simulator-derived metrics for console skills are already part of the da Vinci platform. The contribution of ROBECS One lies in the organization of these elements into a curriculum. Simulator training is combined with procedure-based simulation in alternating operator roles and with dry-lab training on synthetic organ models. Furthermore, four of the six proposed modules (system setup, bedside assistance, whole-procedure performance and non-technical skills) address competencies that are not captured by simulator metrics and require direct observation or dedicated assessment tools. In this pilot, training covered console skills, procedure-based simulation and dry-lab tasks (Modules 3–5); Modules 1, 2 and 6 were derived from the literature to complete the framework and will be implemented and assessed in future iterations. As the benchmarks are defined at module level, they could in principle be applied to other robotic platforms. Table 2 further matches each type of skill to a suitable assessment method, from simulator-derived metrics for psychomotor console skills to direct-observation checklists and rating tools for safety-critical and team-based competencies. Finally, the curriculum was designed to fit within the protected working hours of a general surgery department during a limited period of equipment availability. This reflects the conditions reported by many surgical trainees in Europe, where on-site simulator access during working hours, certified robotic trainers and dedicated training time remain limited [27, 28].

Building on this alignment with existing frameworks, we propose the ROBECS One benchmark structure not as a competing framework, but as a pragmatic, locally implementable operationalization of these broader consensus principles — translating general proficiency-based recommendations into concrete, module-specific benchmarks that can be piloted, iteratively refined, and, pending formal validation, integrated into existing multimodal curricula such as RoSTraC or the international Delphi-based framework [22]. Beyond curriculum design, there is a broader gap in how autonomy is granted in robotic surgery. Even with automatically logged console data, faculty and trainees frequently misjudge how much of a case a trainee performs: faculty overestimate the console time they grant, novices underestimate their own participation, and both groups’ expectations diverge from the recorded data [29]. Survey data reinforce this: most general surgery residents report that an attending’s own experience, rather than their demonstrated skill, determines console time, and roughly half report dissatisfaction with their robotic training as a result [6]. Asked directly what drives entrustment, attendings and residents diverge further: attendings cite resident skill and reputation, residents cite attending confidence and time pressure [30]. Entrustment decisions of this kind are increasingly recognized as central to safe and effective clinical training, since they mark the point at which a trainee is trusted to act with a defined level of supervision [26]. By generating objective, simulator-derived performance data ahead of clinical exposure, structured curricula such as ROBECS One offer a potential evidence base for these entrustment decisions, and could thereby complement subjective, attending-dependent judgment with benchmark-driven, defensible decisions about when a trainee is ready for greater autonomy, at the console and ultimately in the operating room.

## Strengths and limitations

This study addresses an important and timely gap in surgical education by applying a structured, proficiency-based framework to robotic simulation training. The curriculum was successfully delivered to both advanced and novice robotic surgeons, demonstrating that a structured, multimodal robotic curriculum can be feasibly delivered within existing clinical working hours. Training combined virtual reality simulation, procedure-based simulation, and dry-lab practice, allowing coverage of technical skills across a range of task complexity rather than isolated console drills alone. However, several limitations should be acknowledged. The sample size was small and derived from a single center in Switzerland, limiting generalizability. The study design was descriptive and did not include formal assessment of learning outcomes, skill transfer or clinical entrustment. Additionally, the proficiency benchmarks proposed here were developed conceptually after the training had been completed and were not formally validated within this pilot. Participants were classified as advanced or novice based on independent console experience rather than a predefined robotic case volume, which limits comparability with other studies. Participant feedback was collected retrospectively as unstructured written comments without pre- and post-training questionnaires, and acceptability was not assessed quantitatively. The two-week duration was determined by the availability of the robotic system, and retention of skills over time was not assessed. Finally, feedback collected by a member of the study team may have been subject to recall and social desirability bias, and no control group was included.

## Future directions

Building on this feasibility work, future research should focus on collecting simulator-derived performance data, as well as formally validating the proficiency benchmarks proposed in Table 2, including assessment of inter-rater reliability for GEARS-based scoring and correlation with subsequent operative performance. A range of objective and subjective assessment tools, from simulator-derived metrics to structured rating scales, have already been catalogued for robotic surgery training and could inform the choice of evaluation methods for these benchmarks [31, 32]. A further avenue would be to examine whether these simulator-derived benchmarks are predictive of real-world entrustment decisions, such as the console time and operative autonomy trainees are subsequently granted. Future iterations should include structured pre- and post-training questionnaires assessing participants’ confidence, perceived competence and satisfaction with the training.

Multicenter studies are also needed to evaluate the transferability of this curriculum and its impact on clinical performance in the operating room. Given the close structural alignment between this curriculum and the recently published international consensus framework for gastrointestinal robotic training [22], future work could explore testing or adapting elements of that broader curriculum within this simulation-based pilot, contributing local implementation data to its ongoing iterative development.

The integration of structured, benchmark-driven curricula into longitudinal robotic training programs may facilitate a more standardized and proficiency-driven approach to surgical education.

## Conclusion

This pilot study demonstrates that a structured, simulation-based robotic training curriculum can be feasibly implemented within a clinical training environment. Building on this feasibility, we propose a preliminary set of proficiency benchmarks, matched to the technical and non-technical skills required at each training stage, as a first step toward benchmark-driven progression. These benchmarks require formal validation in future, larger-scale studies, but may help inform both standardized robotic training pathways for general surgery trainees, and how operative autonomy and entrustment are granted in robotic surgery, complementing seniority and subjective impression with demonstrated, measurable competence.

## Data Availability

No datasets were generated or analysed during the current study.

## Abbreviations

PBP: Proficiency Based Progression
GEARS: Global Evaluative Assessment of Robotic Skills
RoSTraC: Robotic Surgery Training Curriculum
ERUS: European Robotic Urology Standard
ROBECS: Robotic Educational Curriculum for Surgeons
VR: Virtual Reality
EKNZ: Ethikkommission Nordwest- und Zentralschweiz
BASEC: Business Administration System for Ethics Committees

## Declarations

### Generative Artificial Intelligence statement

The generative Artificial Intelligence tool Claude Sonnet 5 was used for language editing and proofreading of the final version of the manuscript. The authors reviewed and verified the accuracy and integrity of all AI-assisted output. All intellectual content, including ideas, research design, and data analysis was conducted solely by the authors.
